# Adolescent Self‐Inserted Rectal Foreign Body Removed Manually With Topical Anesthetic Only

**DOI:** 10.1002/ccr3.72288

**Published:** 2026-03-11

**Authors:** Takuto Shimada, Shigeki Tsuboi

**Affiliations:** ^1^ Department of Surgery Ogaki Municipal Hospital Gifu Japan; ^2^ Department of Emergency and Critical Care Medicine Ogaki Municipal Hospital Gifu Japan

**Keywords:** adolescent, emergency medicine, manual extraction, rectal foreign body, topical anesthesia

## Abstract

Adolescent cases of self‐inserted rectal foreign bodies are rare. Thorough CT assessment to exclude perforation is essential. Manual extraction using only topical anesthesia with patient cooperation and voluntary muscle control can be safely performed in selected asymptomatic cases.

## 1 Clinical Image Text

**FIGURE 1:**
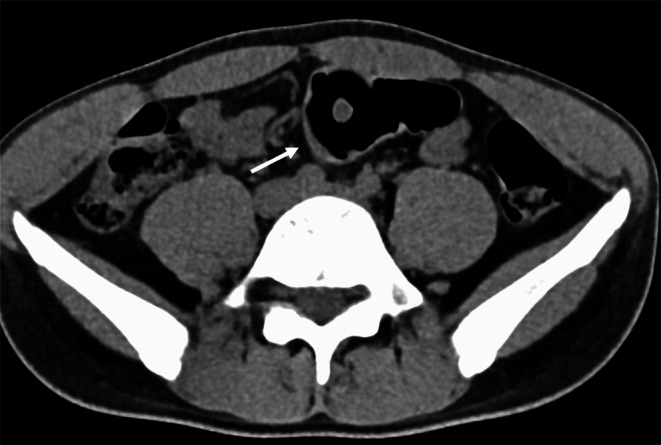
Axial contrast‐enhanced CT showing a cylindrical intrarectal foreign body (arrow) without surrounding inflammation or free air.

**FIGURE 2:**
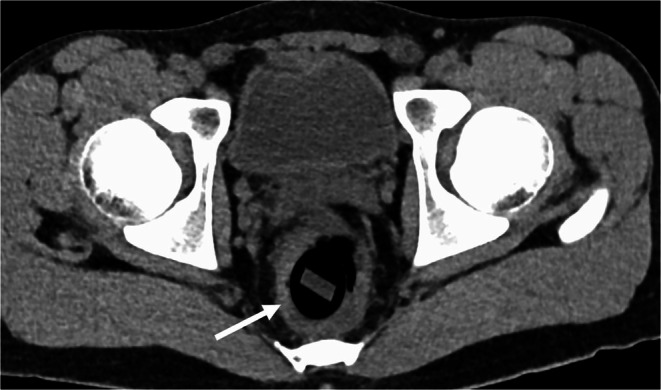
Another level of axial contrast‐enhanced CT showing a cylindrical intrarectal foreign body (arrow) without surrounding inflammation or free air.

**FIGURE 3:**
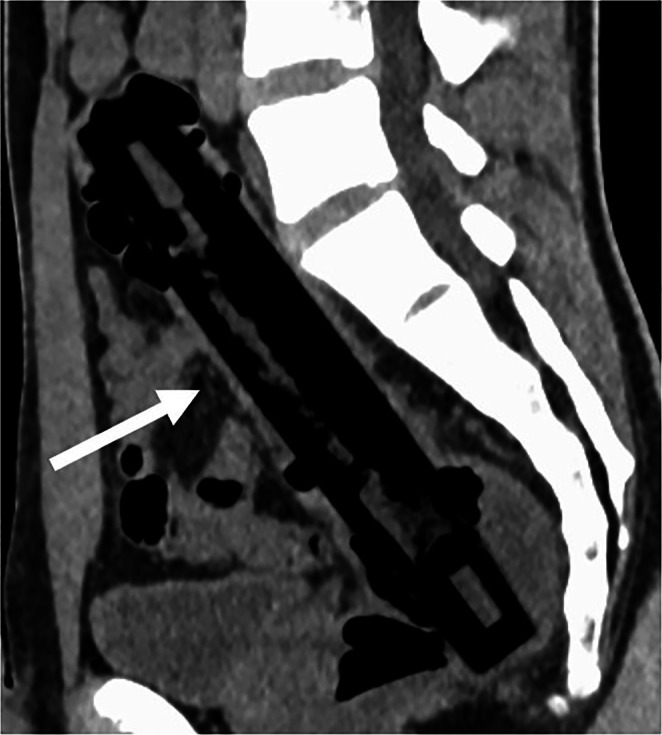
Sagittal CT demonstrating the long‐axis configuration of the object (arrow).

A 15‐year‐old boy presented 9 h after self‐insertion of a cylindrical plastic marker (diameter 21.8 mm, length 140.8 mm) into the rectum for erotic stimulation. He remained asymptomatic without abdominal pain, bleeding, or fever. Physical examination was unremarkable. Digital rectal examination confirmed that the distal tip was reachable at the fingertip without mucosal injury. CT demonstrated a smooth cylindrical object in the rectum without perforation, perirectal fat stranding, free air, or fluid (Figures 1, 2, 3). The extracted object is shown in Figure S1.

The object was extracted in the lithotomy position using a two‐finger grasp and traction after application of 2% lidocaine jelly only. Adult cases often require regional or general anesthesia to overcome sphincter spasm reported in up to 60%–70% of cases [1], however, in this case, the patient was instructed to perform straining, which allowed the foreign body to pass the anal verge. Interestingly, the absence of spinal or regional anesthesia was advantageous in this case, as voluntary contraction and relaxation could be utilized during extraction.

Following extraction, 1 h observation and a repeat contrast CT were performed to definitively exclude delayed perforation or occult mucosal injury to ensure maximum safety in accordance with our institutional clinical practice.

**FIGURE 4:**
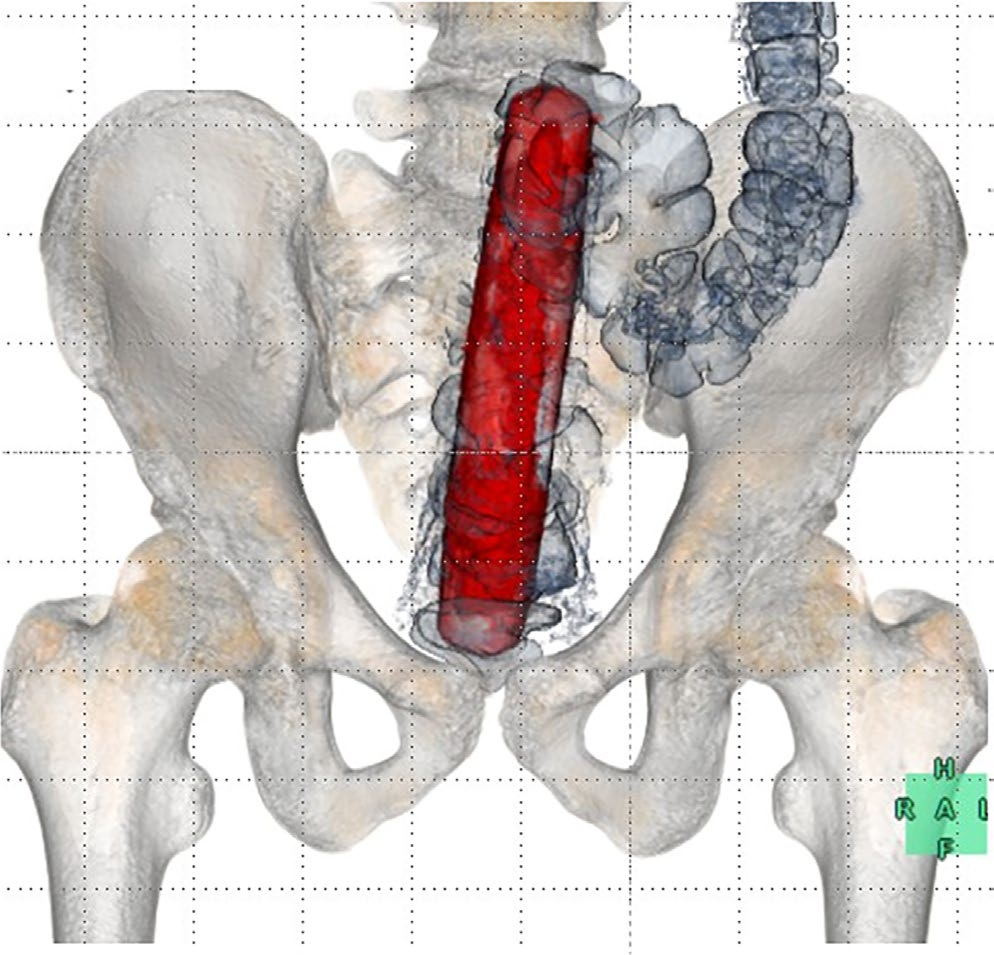
Three‐dimensional reconstruction confirming the intrarectal location, orientation, and shape of the foreign body.

Three‐dimensional reconstruction was valuable not only for identifying the object's orientation but also for confirming its smooth structural configuration and spatial relationship with the pelvic walls [2]. This comprehensive morphological assessment enhanced procedural safety by allowing us to anticipate the extraction trajectory (Figure 4).

## Author Contributions

**Takuto Shimada:** conceptualization, methodology, resources, writing – original draft, writing – review and editing. **Shigeki Tsuboi:** conceptualization, methodology, supervision.

## Funding

The authors have nothing to report.

## Consent

Written informed consent for publication and image use was obtained from the patient and legal guardian in accordance with ICMJE recommendations.

## Conflicts of Interest

The authors declare no conflicts of interest.

## Supporting information


**Figure S1:** Photograph of the cylindrical plastic marker removed from the rectum.

## Data Availability

No datasets were generated or analyzed for this study. All clinical information is contained within the manuscript.
