# Supplementary figures and images for: Adolescent Self‐Inserted Rectal Foreign Body Removed Manually With Topical Anesthetic Only
Source: Clin Case Rep. 2026 Mar 11;14(3):e72288. doi: 10.1002/ccr3.72288 (PMC13093348; doi:10.1002/ccr3.72288)

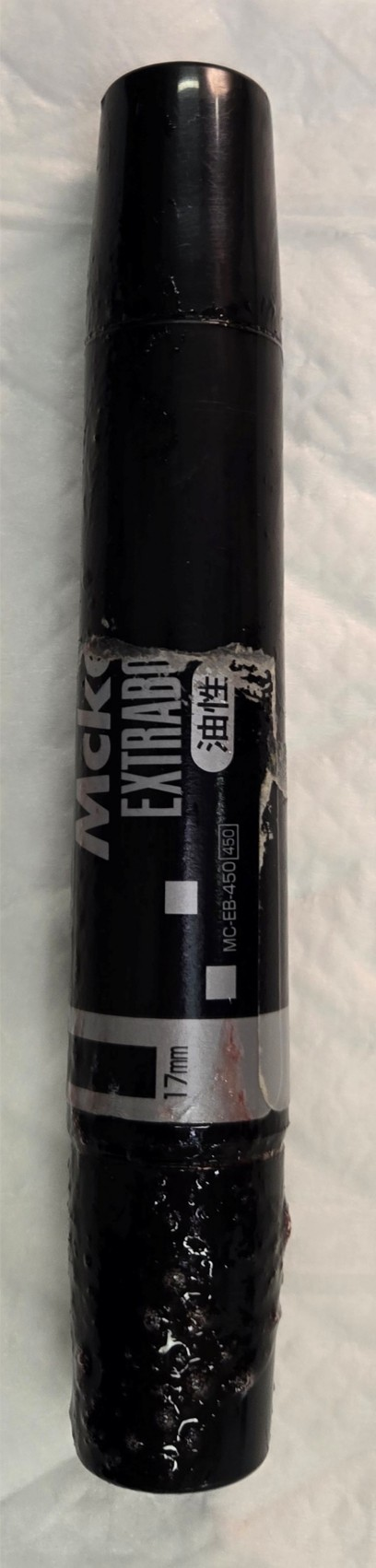

Supplement: Supplementary file 1 — Figure S1: Photograph of the cylindrical plastic marker removed from the rectum. [file CCR3-14-e72288-s001.tiff]
